# DNA Methylation Is a Main Key for Bacteria-Related Colon Carcinogenesis

**DOI:** 10.3390/microorganisms9122574

**Published:** 2021-12-13

**Authors:** Iradj Sobhani

**Affiliations:** 1 Department of Gastroenterology Henri Mondor Hospital APHP, 94010 Créteil, France; iradj.sobhani@aphp.fr; 2 EC2M3-EA7375, Université Paris-Est Créteil, 94010 Créteil, France

Colorectal cancer (CRC) is the second most common cause of cancer deaths in men and women combined [1,2]. Colon-tumor growth is a multistage process, resulting from the accumulation of spontaneous mutations and epigenetic events that silence tumor-suppressor genes while activating oncogenes [3]. Environmental factors are considered to be the primary contributors to somatic gene alterations, thereby accounting for the increased incidences of CRC in Western countries [4].

Only a small number of CRCs are hereditary. These concern individuals with a heterozygous adenomatous polyposis coli (APC) gene deficiency, because they develop a condition called familial polyposis (FAP), which likely evolves into CRC depending on age. In addition to germline mutations, the FAP condition accurately illustrates the critical role played by the environment in the development of additional mutations through other genes including Kras, P53, etc. [5]. Moreover, a second form of hereditary colon cancer caused by defects in mismatch repair or the MMR system, known as Lynch syndrome or human no polyposis colon cancer (HNPPCC), is characterized by numerous mutations, resulting in the development of sporadic CRC. Notably, silencing genes through hypermethylated DNA could be identified in these hereditary syndromes [6]. 

It has been known for several decades that DNA methylation likely regulates gene expression patterns, with CpG sites at promoter regions displaying hypermethylation, whereas other CpGs undergo hypomethylation during aging. Several studies have revealed evidence regarding the relevance of DNA methylation patterns during aging. This has contributed to establishing “epigenetic clock” markers in human tissues which display a robust correlation with age (r > 0.90) [7], also explaining why majority of CRCs occur in elderly individuals. Environmental factors are assumed to enhance DNA demethylation rates. 

In eukaryotes, the predominant type of DNA methylation is 5-methylcytidine (m5C), required for regulating gene expression and development. In prokaryotes, particularly in bacteria, various functions such as protection from foreign DNA invasion are dependent on DNA genome methylation [8]. Epigenetic regulation of virulence factors from restricted methylation systems has been demonstrated in Streptococcus pyogenes pathogens. Overall, genes that encode cell surface proteins, including peptidases that cleave host complements, as well as various proteins essential for adhesion, internalization, and immune evasion phenotypes [9], are dependent on the N6-methyladenine of Streptococcus pyogenes. These observations suggest that bacteria similarly apply the methylation of their genes to enhance their hosting and invasion capacities.

In recent decades, gut microbiota and their metabolites have been recognized as factors that are potentially involved in colon carcinogenesis; they now serve as biomarkers for CRC diagnosis and prognosis [3,4,10]. Given that gut microbiota bacteria are exposed to the same environment as the host they inhabit, the challenging investigation is to further understand **how bacteria likely contribute to carcinogenesis within the host colonic mucosa**. Whether bacteria imprint mutations rather than methylation or carry out both in regard to the host DNA in colonocytes is still under investigation, with putative roles warranting specific approaches.

Based on our research, the majority of sporadic CRC patients display gut microbiota dysbiosis with various genes such as Wif1, SFRP, and SEPTIN involved in the *Wnt* pathway, showing hypermethylation in their promotors, which was reported to be significantly associated with gut microbiota dysbiosis [3,11]. The relationship between dysbiosis, on one hand, and dysmethylation as either the hyper- or hypo-methylation of canonical genes in host colonocyte DNA, on the other, as observed in patients suffering from colon or rectal cancer, was revealed to be associated with age. These interactions require further extensive investigations.

*Wnt* signaling is altered in CRC carcinogenesis, and a strong association between *Wnt* signaling and DNA methyltransferase was demonstrated using mass-spectrometry-based protein analyses. This suggests a direct interaction between β-catenin, a key protein of the *Wnt* pathway, and DNA methyltransferases (DNMT), in a protein–protein system. Interdependent reductions in DNMT and β-catenin signaling by the hypomethylation of several CpG loci do support the role of methylation within epithelial cells during carcinogenesis on the path from normal to cancerous cells. More generally, methylation of the DMNT gene was evaluated in peripheral blood mononuclear cells (PBMCs) in 2453 patients, revealing levels that gradually declined with aging [12]. Accordingly, employing ApcMin mice [13], Laird et al. demonstrated that when genetically and pharmacologically diminishing the DNMT activity, this reduced the number of intestinal neoplasia. After birth, intestinal microbiota organization is accompanied by the maturation of DNA methylation signatures and the transcriptional program in intestinal epithelial cells [14]. The intestinal microbiota modulates DNA methylation, which is likely due to some metabolites such as folate, acting as essential methyl donors.

Chronic inflammatory bowel disease (IBD), which is thought to favor the occurrence of colon cancer, is associated with specific dysbiosis. The latter enhances genome-wide methylation [15], mainly through the deamination of 5′methylcytosines. Given that IBD is primarily diagnosed in young people, 5′methylcytosine activity reduction cannot be explained by the patient age at diagnosis. 

The ten-eleven translocation (TET) enzymes oxidize 5-methylcytosines (5mCs) and promote the locus-specific reversal of DNA methylation. *TET* genes are commonly mutated in various cancers [3]. The role of TET proteins in regulating DNA methylation and transcription constitutes the key question in chronic inflammation-related carcinogenesis [16]. This, together with the deamination of 5′methylcytosines, which is not linked with age, suggests that inflammation changes global DNA, or at least a targeted part of it [17]. Thus, the specific role of virulent bacteria within gut microbiota, in spite of these bacteria being not abundant, becomes increasingly pivotal. 

Tumor growth is presumed to be under regulatory T-cell control, which has been shown to be modulated by gut dysbiosis [18]. T-cell exhaustion is an induced dysfunctional state which is acquired epigenetically. CD8^+^ T-cell exhaustion can be initiated by microorganisms and then become irreversible, thus continuing even after neoplasia has been treated or microorganisms have been eradicated. This means that epigenetic programs of exhaustion in CD8^+^ T-cells continue to be ‘epigenetically scarred’, even after the cause has been controlled. Therapeutic efforts to reverse T-cell exhaustion may require new approaches for increasing the epigenetic plasticity of exhausted T-cells, possibly including microbiota manipulation [19].

In summary, the methylation of DNA in colonic epithelial cells is substantially impacted by dysbiosis. Pro-inflammatory virulent bacteria, the abundance of which is enhanced by Western lifestyles, including diet, constitute the main microbial community involved in gene mutation and methylation. 
